# Complete Genome Sequence for *Treponema* sp. OMZ 838 (ATCC 700772, DSM 16789), Isolated from a Necrotizing Ulcerative Gingivitis Lesion

**DOI:** 10.1128/genomeA.01333-14

**Published:** 2014-12-24

**Authors:** Yuki Chan, Angel P. Y. Ma, Donnabella C. Lacap-Bugler, Yong-Biao Huo, W. Keung Leung, Frederick C. Leung, Rory M. Watt

**Affiliations:** a Oral Biosciences, Faculty of Dentistry, The University of Hong Kong, Pok Fu Lam, Hong Kong SAR, China; b School of Biological Sciences, Faculty of Science, The University of Hong Kong, Pok Fu Lam, Hong Kong SAR, China; c Oral Diagnosis and Polyclinics, Faculty of Dentistry, The University of Hong Kong, Pok Fu Lam, Hong Kong SAR, China; d Bioinformatics Center, Nanjing Agricultural University, Jiangsu, China

## Abstract

The oral treponeme bacterium *Treponema* sp. OMZ 838 was originally isolated from a human necrotizing ulcerative gingivitis (NUG) lesion. Its taxonomic status remains uncertain. The complete genome sequence length was determined to be 2,708,067 bp, with a G+C content of 44.58%, and 2,236 predicted coding DNA sequences (CDS).

## GENOME ANNOUNCEMENT

Spirochete bacteria belonging to the genus *Treponema* are implicated in the etiology of various polymicrobial biofilm infections of the oral cavity. These include periodontal diseases such as periodontitis and gingivitis, as well as endodontic infections (1, 2, 3). Treponemes are also implicated in the etiology of necrotizing ulcerative gingivitis (NUG) (4). Closely related treponeme taxa play pathological roles in anaerobic polymicrobial tissue-destructive diseases in animals (5, 6).

*Treponema* sp. OMZ 838 (37F9HE; ATCC 700772, DSM 16789) was originally isolated by C. Wyss (University of Zurich) in 1998; from microbial biofilm originally sampled from a NUG lesion in the oral cavity of a 31-year-old Chinese male from northeast China (patient code 37 [7]). This strain is unreported in the scientific literature, but the patient group has been previously described (7, 8). It was originally deposited in the ATCC under the name “*Treponema vincentii*,” which has no official standing in taxonomy (2).

*Treponema* sp. OMZ 838 was obtained directly from C. Wyss and was cultured anaerobically in tryptone-yeast extract-gelatin-volatile fatty acids-serum (TYGVS) medium (9). Genomic DNA was purified (QIAamp DNA minikit; Qiagen, Germany); and sequencing was performed on a 454 Life Sciences GS junior system (Bioinformatics Center, Nanjing Agricultural University, Jiangsu, China). An initial shotgun library generated 238,378 reads, and a subsequent 8-kbp span paired-end library yielded 124,329 reads. The combined libraries generated sequencing data with an average of 56× coverage. The *de novo* assembly (454 Newbler version 2.7) yielded 23 large contigs with an *N*_50_ contig size of 561,149 bp in 4 scaffolds. Gaps were closed by PCR and Sanger sequencing.

The *Treponema* sp. OMZ 838 genome is 2,708,067 bp in length, with a G+C content of 44.58%. Sequence data were annotated using the PGAAP pipeline of the NCBI, following the best-placed reference protein set (GeneMarkS+ version 2.7). The complete genome has 2,236 coding sequences (CDS), with 63 pseudogenes, 47 tRNA genes, and two copies of the 5S, 16S, and 23S rRNA gene cluster.

Of note, the genome encodes a major surface protein (MSP) homologue of 492 amino acids (locus tag JO41_03600), which is implicated in various pathological activities (10–13). It putatively comprises three domains analogous to those previously identified in MSP from *Treponema denticola* (13). This protein shares 64.9% and 67.5% amino acid (aa) identity, respectively, with the MSP homologues encoded by “*Treponema vincentii*” ATCC 35580 (511 aa; locus tag TREVI0001_2098 and accession number EEV20349.1), and *Treponema medium* ATCC 700293^T^ (508 aa; locus tag HMPREF9195_00983; accession number EPF29199.1). We envisage that the *Treponema* sp. OMZ 838 (“*Treponema sinensis*”) genome sequence reported here will significantly aid the clarification of taxonomic, phylogenetic, and pathobiological issues concerning treponemes found in oral and nonoral niches within humans and higher animals.

### Nucleotide sequence accession number.

The *Treponema* sp. OMZ 838 genome sequence was deposited in GenBank under the accession number CP009227.
